# Morphological characterization, molecular identification, and metabolic profiles of two novel isolated bamboo mushrooms (*Phallus* sp.) from Thailand

**DOI:** 10.1371/journal.pone.0307157

**Published:** 2024-10-24

**Authors:** Sirilak Chumkiew, Mantana Jamklang, Chompunoot Wangboon, Watsana Penkhrue, Napaporn Mangmee, Kanyapak Sakheatkarn, Pongsakorn Martviset, Pathanin Chantree, Salisa Chaimon, Bumpenporn Sanannam, Malinee Thanee, Manida Suksawat, Jutarop Phetcharaburanin

**Affiliations:** 1 School of Biology, Institute of Science, Suranaree University of Technology, Nakhon-Ratchasima, Thailand; 2 School of Preclinical Sciences, Suranaree University of Technology, Nakhon-Ratchasima, Thailand; 3 Department of Preclinical Science, Faculty of Medicine, Thammasat University, Pathum Thani, Thailand; 4 Thammasat University Research Unit in Nutraceuticals and Food Safety, Thammasat University, Pathum Thani, Thailand; 5 Department of Pathology, Faculty of Medicine, Khon Kaen University, Khon Kaen, Thailand; 6 Khon Kaen University International Phenome Laboratory, Khon Kaen University Science Park, Innovation and Enterprise Affairs, Khon Kaen University, Khon Kaen, Thailand; 7 International College, Khon Kaen University, Khon Kaen, Thailand; 8 Systems Biosciences and Computational Medicine, Faculty of Medicine, Khon Kaen University, Khon Kaen, Thailand; ICAR - Central Tobacco Research Institute, INDIA

## Abstract

The well-characterized edible and medicinal bamboo mushroom is *Phallus indusiatus*, a Chinese bamboo mushroom with long white indusium (skirt). To date, scientists have found more than five species of bamboo mushrooms in Thailand, containing bamboo mushrooms with long white, short white, and several colored skirts. Still, most of them are unidentified species and lack metabolic profile data. Hence, this study aims to evaluate the species of the long white-skirt Chinese bamboo mushroom-like (CH-isolate) and short white-skirt (TH-isolate) bamboo mushrooms isolated from a local farm in northern Thailand. External morphology and molecular identification were used to identify the species. Nutritional and metabolic studies were conducted to determine the nutrients and metabolites from both isolates. Our morphological and evolutionary phylogenetic analyses suggested that CH- and TH-isolates were different species. Interestingly, the CH-isolate, which has a similar morphology to *P*. *indusiatus*, clearly demonstrated the difference in species. In addition, the nutritional and metabolomic analysis revealed that CH- and TH-isolate contain different nutritional constituents and metabolic profiles. Our study reports the two new species of bamboo mushrooms that were suspected to be found in Thailand and their metabolic profiles that could be beneficially used in further studies. However, definitive confirmation of the novel species will be made in the future.

## Introduction

Bamboo mushroom is an edible and medicinal fungus that is an ingredient of traditional meals in several Asian countries, including China, Japan, India, Vietnam, and Thailand. Even though it is popular in some countries, this mushroom can be found on all continents, especially in the forests of the tropical zones in Asia, Africa, America, and Australia [1, 2]. Most of them belong to the phylum of Basidiomycetes, the family Phallaceae, the genus of *Phallus* or *Dictyophora*, which is characterized by a cylindroid stalk with a net-like indusium or skirt hanging from a bell-shaped cap [3]. Among them, *Phallus indusiatus* (*Dictyophora indusiata*) (Vent.). or Chinese bamboo mushroom, has been widely studied [3, 4]. The external morphology of *P*. *indusiatus* is a rough, rounded cone-shaped cap with a long white skirt [4, 5]. In Thailand, more than five species have been reported, including the long white skirt bamboo mushroom originating from a Chinese strain (referred to *P*. *indusiatus*), shorted-white skirt bamboo mushroom (such as *P*. *ultraduplicatus*, *P*. *chiangmaiensis*, and *P*. *merulinus*), orange skirt bamboo mushroom (*P*. *multicolor* var. *lacticolor*), red skirt bamboo mushroom (*D*. *rubrovolvata*), and yellow skirt bamboo mushroom (*P*. *multicolor*) [6, 7]. However, most of the reported bamboo mushrooms are characterized by morphological-based procedures that can separate only major distinctive structures, but only a few molecular identifications and chemical taxonomy have been launched [3, 8]. Kang et al. [9] reported that the chemical taxonomy relying on secondary metabolites exhibited greater accuracy than sequence-based methods. Previous studies used various methods for identifying the secondary metabolite, such as HPLC fingerprinting combined with stoichiometric network analysis [10] and the NMR metabolomics method; NMR metabolomics allowed for a faster and less time-consuming identification process, which aligns with the demands of large-scale production quality control [11]. This taxonomic identification method based on specific fungal metabolites effectively overcomes the limitations associated with the low accuracy of traditional methods [8].

From all identified bamboo mushrooms, the white skirt bamboo mushrooms are edible, but the colored skirt bamboo mushrooms are mostly inedible. So, these long and short white skirt bamboo mushrooms are more attractive for cultivation and application as nutraceuticals and food supplements [12]. Edible bamboo mushrooms have a wide range of effects, such as anti-inflammation [7, 13], immunostimulation [14], wound healing and collagen stimulation [7], anti-oxidation [5], and anti-cancer [15, 16] activities. The well-characterized bioactive constituents are polysaccharides, but most of the bioactive constituents remain unknown [5, 14]. In *P*. *indusiatus*, carbohydrates share approximately 47% of the dry weight with 38% soluble polysaccharides such as β-(1→3)-D-glucan [14]. Meanwhile, crude protein and fiber are about 6% and 29%, respectively [17]. Furthermore, essential amino acids and beneficial minerals such as vitamin E, β-carotene, ascorbic acid, thiamine, riboflavin, nicotinic acid, phosphate, and calcium are rarely found [17–19].

For that reason, the principal objective of this study is to identify the species of collected bamboo mushrooms and evaluate their bioactive constituents using metabolomic analysis. In this present study, the white skirt bamboo mushrooms (both long and short skirts) cultured in farms in Thailand were investigated. The conventional morphological-based identification was used to separate the collected bamboo mushrooms into two distinguished groups. The long white-skirt Chinese bamboo mushroom-like (*P*. *indusiatus*) was named CH-isolate, while the short white-skirt bamboo mushroom was named TH-isolate. Moreover, molecular characterization was applied to identify the species of the mushroom as well as the evolutionary revolution. Additionally, the metabolomic analysis was used to determine the metabolites of these mushrooms that could be used as a source of bioactive compounds for further investigations.

## Materials and methods

### Bamboo mushroom sample collection

*Phallus* sp. samples were obtained from Ban Ko Ruak village, Mae Mo district, Lampang provinces, northern Thailand, where these mushrooms were naturally found and could be continuously farmed. Initially, the two bamboo mushrooms with external morphological differences, including CH- and TH-isolate, were collected separately. All stages of both mushrooms, including eggs, immature fruiting bodies, and mature fruiting bodies, have been collected for species identification.

### Morphological characterization of bamboo mushrooms

The freshly collected bamboo mushrooms (five samples of each isolate) were observed based on morphological characters. The size of the eggs, fruiting bodies, stalks, caps, annuli, and volva were measured in centimeters. The shape, color, and other characters were recorded. The morphological identification was identified following the guidelines of Gunasekaran and Senthilarasu [20, 21], Yadav et al., [22, 23], and Izumitsu [24].

### Isolation of bamboo mushroom genomic DNA

The fruiting bodies of CH- and TH-isolate bamboo mushrooms (five samples of each isolate) were used for genomic DNA isolation using a rapid and straightforward method described by Izumitsu [24] with a few modifications. Briefly, a minute of mycelium from the fruiting body was picked using a sterile toothpick and then added to the microcentrifuge tube containing 100 μl of TE buffer, pH 8.0 and microwaved for 1 min at 600W twice. The tube was cooled down, kept at -20 °C for 10 min, and centrifuged at 10,000 *× g* for 5 min. The supernatant was collected and used as a template for PCR amplification.

### PCR amplification and molecular cloning of ITS-PCR products

PCR amplification procedure and specific primers were obtained from previous studies [7, 23]. The primer set called D03 (Fw: 5ʹ- TGC CTG TTT GAG TGT CGT GA-3ʹ and Rv: 5ʹ-ACG GAC GAC GCA AGA CTT AT-3ʹ) and D04 (Fw: 5ʹ-GGA AGT AAA AGT CGT AAC AAG G-3ʹ and Rv: 5ʹ-ACG GAC GAC GCA AGA CTT AT-3ʹ) were designed from *Dictyophora indusiata* (*Phallus indusiatus*) internal transcribed spacer-1 (ITS-1) and -2 (ITS-2), respectively. The *Taq* DNA polymerase-based PCR reactions (ThermoScientific, Lithuania) were prepared according to manufacturing instructions and amplified in the PCR thermocycler (Mastercycler^®^ nexus, Eppendorf, Germany) with initial denatured at 94 ºC, for 5 min followed by 35 cycles of denaturing at 94 ºC, for 1 min, annealing at 55 ºC, for 1 min, and extension at 72 ºC, for 2 min, and one cycle of final extension at 72 ºC, for 10 min. The PCR products were size-separated in 2% agarose gel electrophoresis and observed under a UV transilluminator. The PCR products were extracted from agarose gel by using a gel extraction kit (GeneJET Gel Extraction Kit, ThermoScientific, USA) and ligated with pGEM-T easy vector (Promega, USA), then transformed into XL-1 blue *Escherichia coli* competent cells as previously described [24]. The positive transformants were determined by direct colony PCR, and the plasmid DNAs were isolated using the QIAprep Spin Miniprep Kit (QIAGEN, Germany). Plasmid DNAs were sequenced using the DNA sequencing service of Solgent (Republic of Korea). The sequences were multiple aligned with the existing GenBank database using Clustal Omega (https://www.ebi.ac.uk/Tools/msa/clustalo/) [25], and the evolutionary phylogenetic analyses were generated by using MEGA version XI (https://www.megasoftware.net) [26].

### Phylogenetic analysis

The ITS sequences of CH- and TH-isolate from D03 (CH0306 and TH0306) and D04 (CH0407 and TH0406) primer sets aligned with related *Phallus* spp. including *P*. *atrovolvatus*, *P*. *aureolatus*, *P*. *campanulatus*, *P*. *chiangmaiensis*, *P*. *cinnabarinus*, *P*. *coronatus*, *P*. *denigricans*, *P*. *dongsun*, *P*. *echinovolvatus*, *P*. *flavocostatus*, *P*. *fuscoechinovolvatus*, *P*. *hadriani*, *P*. *haitangensis*, *P*. *impudicus*, *P*. *indusiatus*, *P*. *lutescens*, *P*. *luteus*, *P*. *mengsongensis*, *P*. *merulinus*, *P*. *multicolor*, *P*. *purpurascens*, *P*. *rubrovolvatus*, *P*. *rugulosus*, *P*. *serratus*, *P*. *squamulosus*, *P*. *ultraduplicatus*, and distinctive species *Mutinus albotruncatus*, by using muscle alignments and the evolutionary history was generated by using the Neighbor-Joining method. The percentage of replicate trees in which the associated taxa clustered together in the bootstrap test (1000 replicates) are shown as the branches. The tree is drawn to scale, with branch lengths in the same units as those of the evolutionary distances used to infer the phylogenetic tree. This analysis involved 31 nucleotide sequences. All ambiguous positions were removed for each sequence pair (pairwise deletion option).

### Nutritional value

Mushroom samples of both CH- and TH-isolate were analyzed for chemical compositions (e.g., protein, fat, carbohydrates, fiber and ash) according to the method of the Association of Official Analytical Chemists (2019) [27]. The crude fiber content of samples was estimated by FIBRETHERM^®^ method; the crude protein content (N × 4.38) of samples was estimated by the Kjeldahl method; the crude fat was estimated by extracting a known weight of powdered mushroom samples with petroleum ether, using Soxhlet apparatus; the ash content and the moisture content were determined by incineration at 600 °C and 107 °C, respectively. Total carbohydrate and total energy were calculated by using the following equations: total carbohydrates = 100 –(g moisture + g protein + g fat + g ash) and total energy (kJ) = 17 × (g protein+ g carbohydrate) + 37 × (g lipid), respectively [28].

### Sample preparation for NMR

Nine samples of each CH- and TH-isolate were used for this analysis. The 20 mg of each sample was soluble with D_2_O (pH 7.0) containing 0.1% (w/w) trimethylsilylpropanoic acid (TSP). The sample was passed through a 0.20 μm filter (Corning, USA), and the supernatant was transferred into a 5 mm NMR tube.

### ^1^H NMR data acquisition

^1^H NMR analysis was performed using Bruker AVANCE III 400 MHz spectrometer (Bruker Corporation, USA) at 300 K. All samples were analyzed using standard 1-dimension pulse sequence (recycle delay-90°-t1-90°-tm-°-acquisition) with t1 and to 3 ms, tm to 10 ms, and 90° pulse to 10 μs in 64 scans [29].

### Spectral data processing

The phase and baseline of all NMR spectra were adjusted in MATLAB software, and the TSP peak was set to 0 ppm. Data points within 0.6–9.0 ppm were kept, and the water peak interval (4.5–5.0 ppm) was removed. The data were calculated using a control spectrum after probabilistic quotient normalization (PQN).

### Metabolite identification and network analysis

Pseudo-two-dimensional spectra were drawn based on preprocessed data using statistical total correlation spectroscopy (STOCSY), indicating the correlation between factors among each chemical shift. The ratio of false positives and the threshold of the correlation factor r were calculated. *The p-value was adjusted with Bonferroni’s* false discovery rate (FDR) correction. The area under peaks was also integrated and used as relative concentration. The relative concentrations of metabolites were analyzed using log2 fold-changes using GraphPad Prism 5 (GraphPad Software, Inc., CA, US). The metabolites with log2 fold-changes were analyzed using Spearman correlation using R programming. The heatmap analysis was performed using MetaboAnalyst (www.metaboanalyst.ca/). The MBROLE2 system is accessible at http://csbg.cnb.csic.es/mbrole2.

## Results

### Morphological differences of CH and TH-isolated bamboo mushrooms

**Fig 1:**
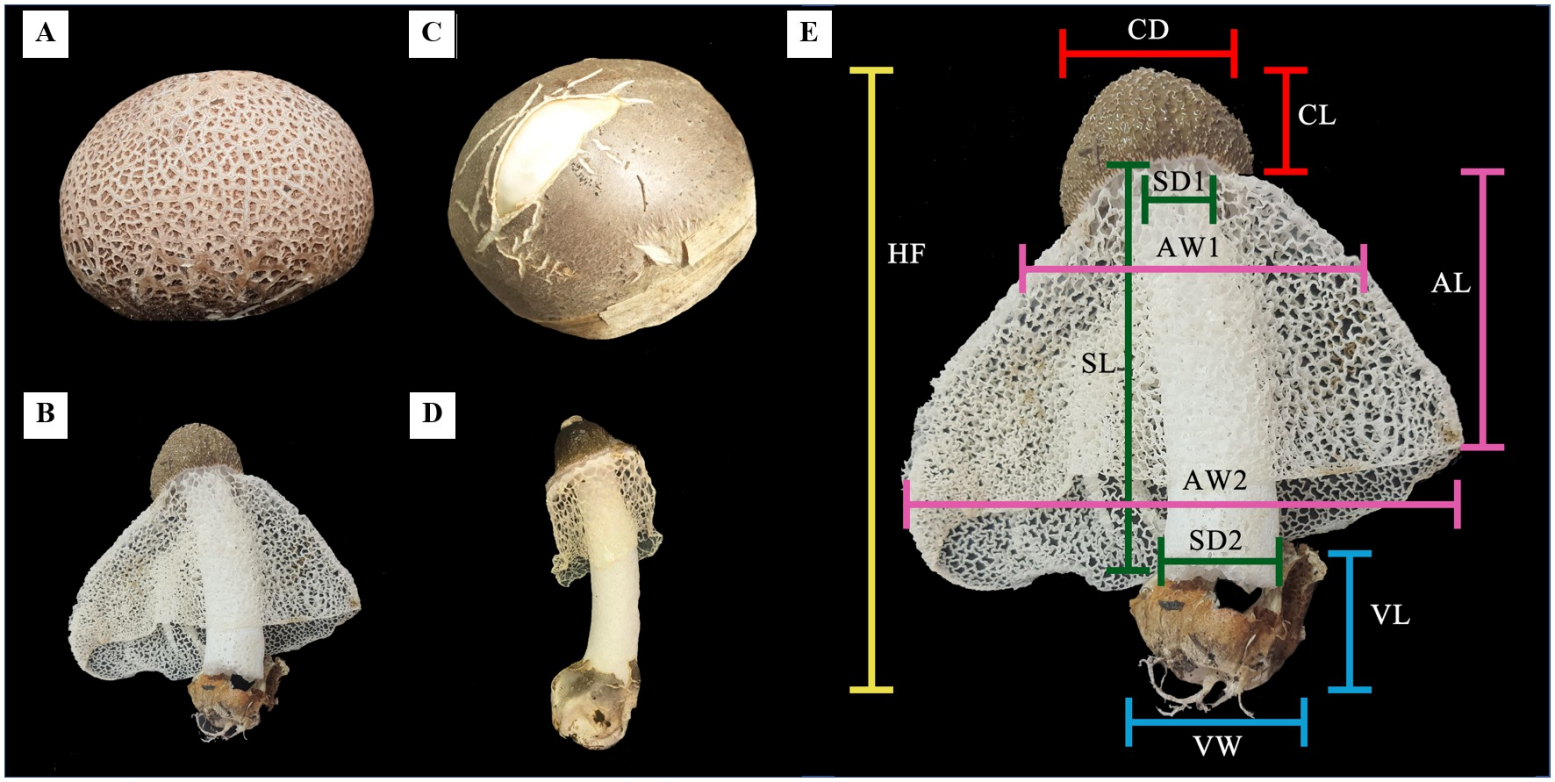
The morphological appearance and differentiated characteristics of CH- and TH-isolated bamboo mushrooms. (A) egg stage of CH-isolate; (B) the fruiting body of CH-isolate; (C) the egg stage of TH-isolate; (D) the fruiting body of TH-isolate; and (E) the identification features of the bamboo mushrooms. The measured parameters of the isolated bamboo mushroom consist of Hight fungi (HF), Cap long (CL), Cap diameter (CD), Stalk long (SL), Stalk diameter (SD) top/bottom, Annulus long (AL), Annulus width (AW) top/bottom, Volva long (VL), and Volva width (VW).

The characteristics of the CH- and TH-isolate were different in several parts. In the egg stage, from external morphology, the CH-isolate illustrated a rough surface (Fig 1A). In contrast, the TH-isolate had smooth skin (Fig 1C), but the size of the eggs was almost similar. The fruiting bodies of CH-isolates (Fig 1B) and TH-isolates (Fig 1D) mostly resembled the measured parameters shown in Fig 1E. On average, CH-isolate’s high fungi (HF) was 13.60 ± 4.81 cm, TH-isolate was 12.90 ± 0.56 cm, and CH-isolate’s cap long (CL) was 2.85 ± 1.34 cm. In comparison, the TH-isolate was 2.42 ± 0.44 cm, the cap diameter of CH-isolate was 4.20 ± 2.41 cm while the TH-isolate was 3.83 ± 0.79 cm, the stalk long (SL) of CH-isolate was 9.60 ± 2.26 cm. In comparison, the TH-isolate was 9.83 ± 1.03 cm, and the stalk diameter at the upper part (SD1) of the CH-isolate was 2.15 ± 0.99 cm. In comparison, TH-isolate was 1.95 ± 0.25 cm, the stalk diameter at the bottom part (SD2) of CH-isolate was 2.70 ± 0.92 cm while TH-isolate was 2.42 ± 0.12 cm, the annulus long (AL) of CH-isolate was 7.95 ± 5.02 cm while TH-isolate was 3.63 ± 0.53 cm. The parameters that demonstrated the different morphology of CH and TH-isolates included the annulus width at the upper part (AW1) of the CH-isolate, which was 11.00 ± 6.36 cm. In comparison, the TH-isolate was 3.40 ± 0.93 cm, the annulus width at the bottom part (AW2) of CH-isolate was 5.25 ± 4.03 cm while the TH-isolate was 3.60 ± 0.91 cm, the vulva long (VL) of CH-isolate was 1.8 ± 0.28 cm. In comparison, the TH-isolate was 2.83 ± 0.13 cm, and the vulva width (VW) of the CH-isolate was 3.35 ± 0.92 cm, while the TH-isolate was 3.35 ± 0.12 cm. Moreover, the fresh weight of the CH-isolate was higher than that of the TH-isolate; the CH-isolate was 23.42 g ± 21.53 g, while the TH-isolate was 13.18 g ± 1.23 g. The other characteristics were observed in the cap, stalk, indusium, resembling roots (or mycelium), and gills. The cap of the CH-isolate was light brown and rounded in shape, while the TH-isolate was darker and coned in shape. The stalks were similar to long cylindroid-shaped, but the TH-isolate was slandered and a little yellower. For the indusium, the CH-isolate had a massive net-like appearance covering all stalks long, while the TH-isolate was smaller. The mycelia and gills were similar and could not be differentiated. All characteristics and measured parameters are indicated in Table 1.

**Table 1:** The characteristics and measured parameters of fruiting bodies of the isolated bamboo mushrooms.

Characteristics	CH-isolate	TH-isolate
**General Appearance**		
Cap color	Light brown	Dark brown
Cap shape	Rounded	Coned
Stalk color	White	Cream
Stalk shape	Long cylindroid	Long cylindroid
Indusium (skirt)	White, long net-like appearance	Cream, short net-like appearance
Resembling roots (mycelium) color	White	Cream
Gills color	Yellow	Yellow
**Average size (centimeters)**		
Hight fungi (HF)	13.60 ± 4.81	12.90 ± 0.56
Cap long (CL)	2.85 ± 1.34	2.42 ± 0.44
Cap diameter (CD)	4.20 ± 2.41	3.83 ± 0.79
Stalk long (SL)	9.60 ± 2.26	9.83 ± 1.03
Stalk diameter (SD) top/bottom	2.15 ± 0.99/2.70 ± 0.92	1.95 ± 0.25/2.42 ± 0.12
Annulus long (AL)	7.95 ± 5.02	3.63 ± 0.53
Annulus width (AW) top/bottom	11.00 ± 6.36/5.25 ± 4.03	3.40 ± 0.93/3.60 ± 0.91
Volva long (VL)	1.8 ± 0.28	2.83 ± 0.13
Volva width (VW)	3.35 ± 0.92	3.35 ± 0.12
Fresh weight	23.42 g ± 21.53	13.18 g ± 1.23

### The evolutionary phylogenetic tree suggested that CH- and TH-isolate are different species

**Fig 2:**
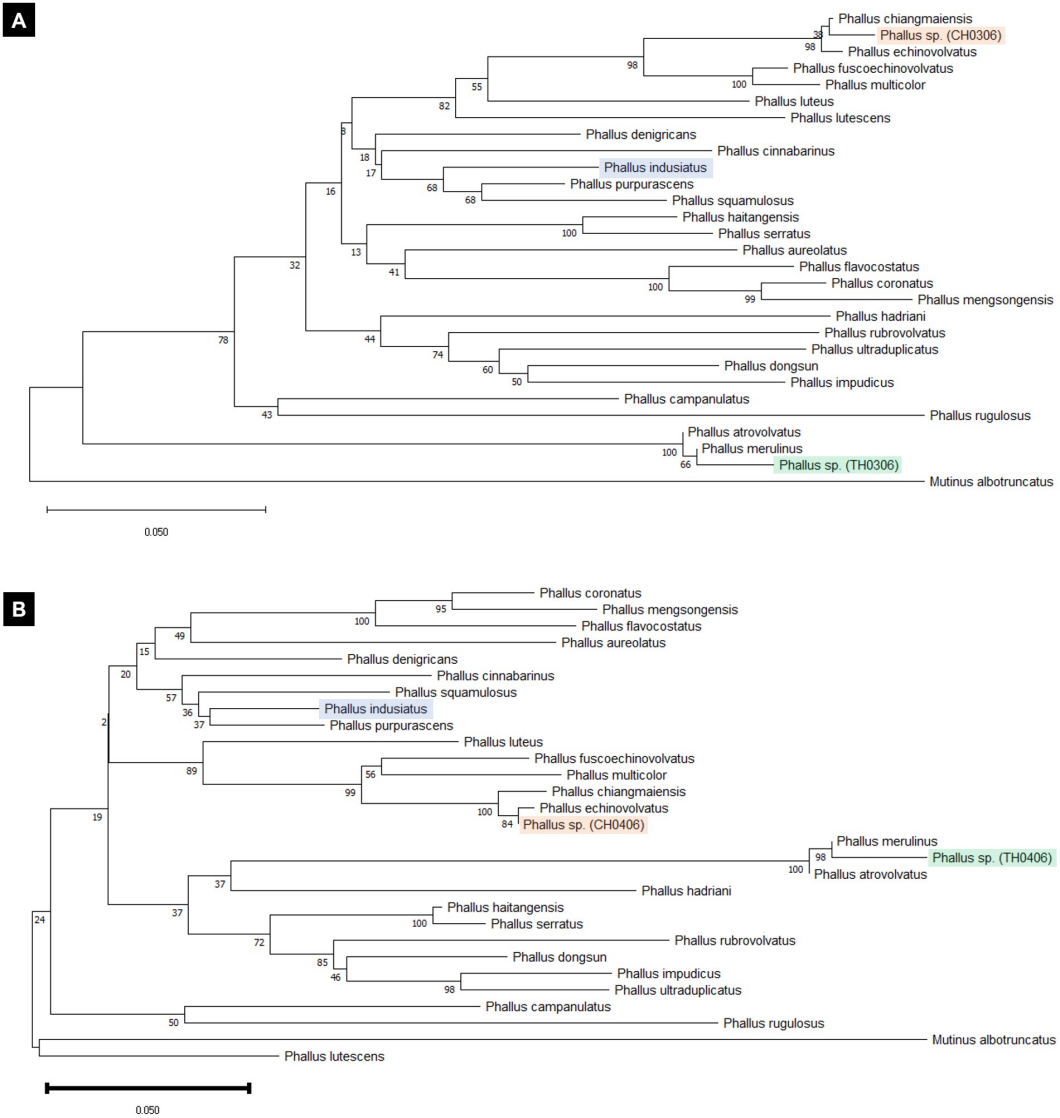
Phylogenetic relationship of isolated bamboo mushrooms (*Phallus* sp.) inferred from ITS sequences of (A) ITS-1 and (B) ITS-2 regions. The number at the significant nodes represents the bootstrap value. CH0306 (LC830479) and CH0406 (LC830481) represented the CH-isolate, and TH0306 (LC830478) and TH0406 (LC830480) represented the TH-isolate.

The molecular phylogenetic analysis is based on ITS-1 (D03 primer set) in Fig 2A. and ITS-2 (D04 primer set) sequences in Fig 2B. The results from both primer sets suggested the same result: the CH-isolate (coded CH0306 for ITS-1 region and CH0406 for ITS-2 region) was well-separated from *P*. *indusiatus* but clustered with *P*. *chiangmaiensis* and *P*. *echinovolvatus*, moreover, they were closely related to *P*. *fuscoechinovolvatus and P*. *multicolor*. On the other hand, the TH-isolate (coded TH0306 for ITS-1 region and TH0406 for ITS-2 region) was well distinguished from the CH-isolate and *P*. *indusiatus* by generating the individual branch with *P*. *merulinus* and *P*. *atrovolvatus*. The gene sequences of bamboo mushroom sample were deposited in the DNA Data Bank of Japan (DDBJ) under accession number as shown in S1 Table.

### Nutritional value

The results of nutritional analyses and estimated energetic value of both CH- and TH-isolate are shown in Table 2. Carbohydrate was found in high levels of both bamboo mushroom samples, and TH-isolate has a higher value of carbohydrate content than CH-isolate, with 67.28 g/100g and 48.99 g/100g, respectively. Protein ranged from 18.88 g/100g in TH-isolate and 31.19 g/100g in CH-isolate. Fiber ranged from 1.60 g/100g in CH-isolate and 8.85 in TH-isolate. Ash ranged from 7.2 g/100g in TH-isolate and 13.10 g/100g in CH-isolate. Fat ranged from 0.34 g/100g in TH-isolate and 1.02 g/100g in CH-isolate. TH-isolate has slightly higher energetic providing than CH-isolate, with 1,477.30 kJ/100g and 1400.80 kJ/100g, respectively (S2 Table.).

**Table 2:** The proximate nutritional value (g/100g) and energetic value (kJ/100g) of bamboo mushroom samples.

Species	Fiber	Fat	Protein	Ash	Carbohydrates	Energy
**TH-isolate**	8.85	0.34	18.88	7.20	67.28	1,477.30
**CH-isolate**	7.60	1.02	31.19	13.10	48.99	1,400.80

Results are expressed on a dry weight basis.

### ^1^H NMR spectra and metabolic constituents of CH- and TH-isolate were distinguishable

**Fig 3:**
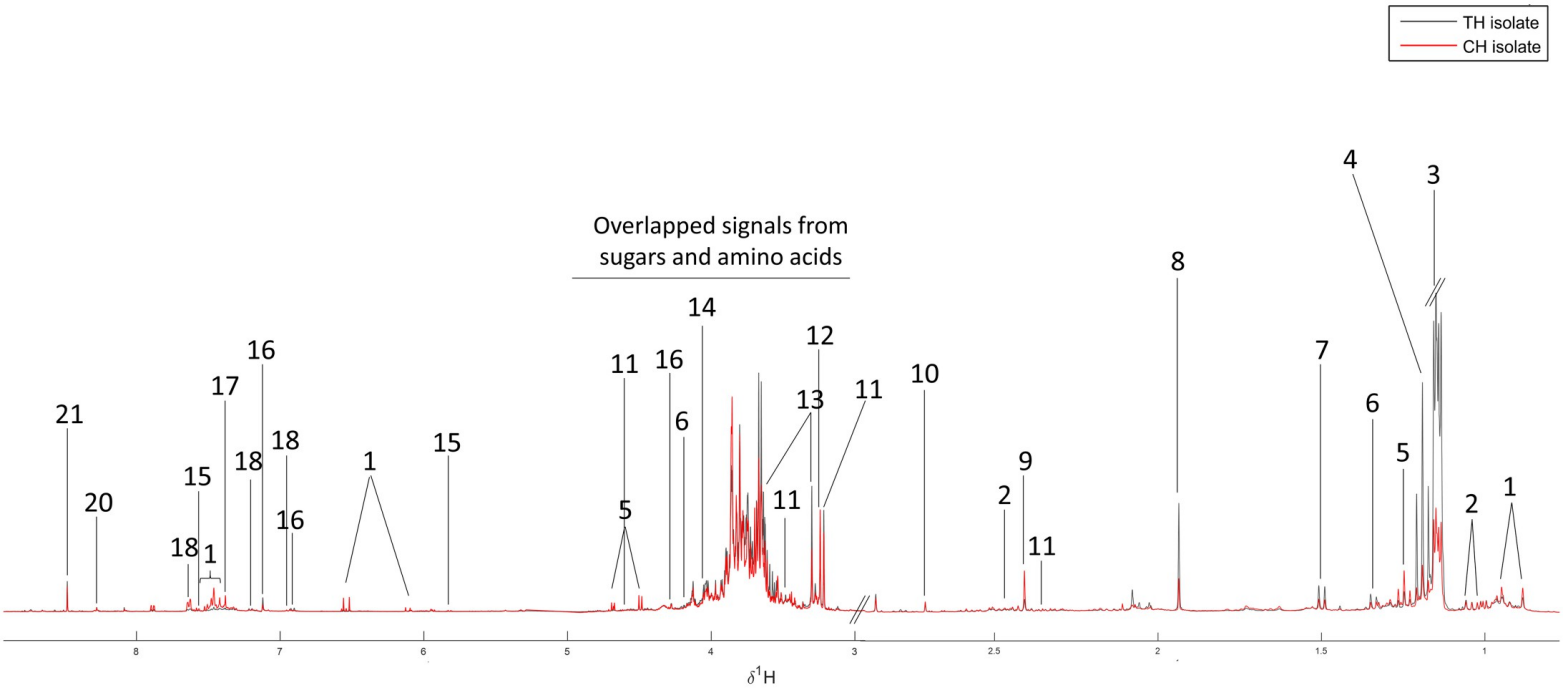
A representative ^1^H NMR spectrum of the aqueous fraction of CH and TH isolated bamboo mushroom extracts. 1, Unknown1; 2, Valine; 3, Unknown2; 4, Ethanol; 5, Unknown3; 6, Threonine; 7, Alanine; 8, Acetate; 9, Succinic-Acid; 10, Dimethylamine; 11, Carnitine; 12, Trimethylamine N-oxide; 13, 3,7-Dimethyluric acid; 14, Glucose; 15, Uracil; 16, Epicathecin; 17, Phenylalanine; 18, Xanthurenate; 19, Histidine; 20, Unknown4; 21, Formate.

CH- and TH-isolate were subjected to verify the metabolic profiles using the ^1^H NMR technique. The peaks were identified by comparison with the chemical shifts of standard components and 2D NMR using ^1^H-^1^H correlation spectroscopy (COSY), heteronuclear multiple quantum coherence (HMQC), and heteronuclear multiple bond coherence (HMBC). A representative ^1^H NMR spectrum of the aqueous fraction of mushroom extract from CH- and TH-isolate showed 21 various metabolites, including valine, ethanol, threonine, alanine, acetate, succinic acid, dimethylamine, carnitine, trimethylamine N-oxide,3,7-dimethyluric acid, glucose, uracil, epicatechin, phenylalanine, xanthurenate, histidine, formate and four unknown metabolites as shown in Fig 3. Interestingly, the unknown 2 metabolites (peak no. 3) abundantly recovered from the TH-isolate but did not exist in the CH-isolate.

The chemical shift of metabolites from CH- and TH-isolate was verified using R^2^ correlation Table 3. The result demonstrated that some metabolites are found in both isolates (R^2^ correlation value close to 1.000), including succinic acid (R^2^ = 0.9839), 3,7-dimethyluric acid (R^2^ = 0.9597), and glucose (R^2^ = 0.9891). On the other hand, the metabolites that were primarily found in CH-isolate but less in TH-isolate were valine (R^2^ = -0.9184), dimethylamine (R^2^ = -0.9550), carnitine (R^2^ = -0.9345), uracil (R^2^ = -0.9749), epicatechin (R^2^ = -0.9829), and unknown 1 (R^2^ = -0.9636). Corresponding to the ^1^H NMR spectrum, unknown 2 was tremendously found in the TH-isolate only (R^2^ = -0.9905).

**Table 3:** The representative data of chemical shifts correlated with metabolites are found in CH and TH-isolated bamboo mushrooms.

NO.	Chemical shift	Metabolite	R^2^ Correlation of CH and TH-isolates
1	0.8847 (s); 0.949 (s); 6.096 (d); 6.519 (d); 7.462 (m); 7.881 (d)	Unknown1	-0.9636
2	1.006 (d); 1.04 (d); 2.26 (m); 3.61 (d)	Valine	-0.9184
3	1.1337 (dd)	Unknown2	-0.9905
4	1.173 (t); 3.618 (q)	Ethanol	-
5	1.229 (t); 4.483 (d); 4.673 (d)	Unknown3	-
6	1.322 (d); 3.611 (d); 4.236 (q)	Threonine	-
7	1.49 (d); 3.751 (q)	Alanine	-
8	1.936 (s)	Acetate	-
9	2.409 (s)	Succinic acid	0.9839
10	2.172 (s)	Dimethylamine	-0.955
11	2.37 (m); 3.217 (s); 3.404 (m); 4.53 (m)	Carnitine	-0.9345
12	3.242 (s)	Trimethylamine N-oxide	-
13	3.3 (s); 3.636 (s)	3,7-Dimethyluric acid	0.9597
14	3.12 (dd); 3.266 (dd); 3.378 (m); 3.514 (t); 3.697 (m); 4.023 (dd);	Glucose	0.9891
15	5.812 (d); 7.565 (d)	Uracil	-0.9749
16	2.771 (dd); 2.894 (dd); 4.295 (m); 6.096 (dd); 6.902 (m); 7.121 (s)	Epicathecin	-0.9829
17	3.081 (dd); 3.394 (dd); 4.023 (dd); 7.322 (d); 7.381 (m)	Phenylalanine	-
18	6.925 (s); 7.18 (d); 7.422 (t); 7.627(d)	Xanthurenate	-0.9962
19	3.12 (dd); 3.266 (dd); 4.023 (dd); 7.122 (s); 7.8768 (s);	Histidine	-0.9763
20	8.276 (s)	Unknown4	-0.9863
21	8.481 (s)	Formate	0.9863

**PCA:** Q^2^ = 0.903**/ OPLSDA:** R^2^X = 0.963, Q^2^Y = 0.994/ p-value = 0.05

### Untargeted metabolomics analysis using PCA and OPLS-DA

**Fig 4:**
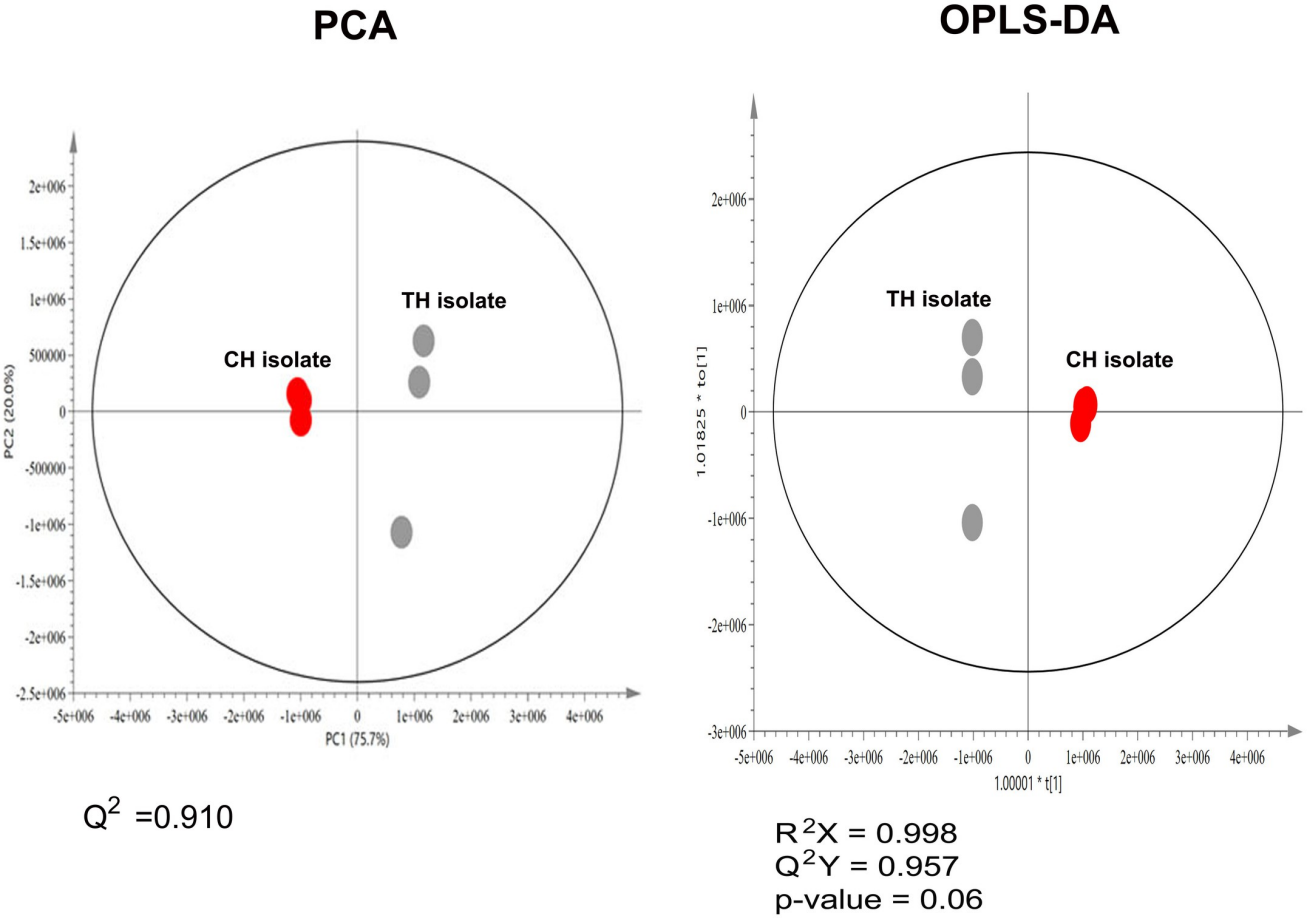
The principal component analysis (PCA) of metabolomics results. (A) PCA score plot between the group of CH- and TH-isolate. (B) The OPLS-DA score plot between the group of CH- and TH-isolate. The data was Par scaling.

From the ^1^H NMR result, a total of 21 metabolic features were observed. The metabolite differences of CH- and TH-isolate were differentiated by employing pattern recognition methods, including unsupervised model Principal components analysis (PCA) and supervised Orthogonal Projections to Latent Structures Discriminant Analysis (OPLS-DA) model. PCA is an unsupervised clustering method that does not require knowledge of the data set and reduces the dimensionality of multivariate data while preserving most of the variance therein [1]. The PCA spots showed significant differences between CH- and TH-isolates, as represented in Fig 4A, with the first two principal components (PC) accounting cumulatively for 91.0% (Q^2^ = 0.910) of the total variance. This result suggested that CH- and TH-isolate were fallen in different species. OPLS-DA is a supervised clustering method that separates the different metabolic profiles based on the group of samples. The OPLS-DA analysis demonstrated more robust clustering discrimination between CH and TH-isolates (R^2^X = 0.998, Q^2^Y = 0.957, *p*-value = 0.06) (Fig 4B). Even though the statistical analysis had insignificant differences, the trend was similar to the PCA analysis. However, when looking at the raw intensity data shown in Table 4, this OPLS-DA insignificant result could be affected by the individual metabolites intensities that showed the differences in only a few metabolites.

**Table 4:** The intensities of all metabolites in CH- and TH-isolate.

Metabolites	CH-isolate	TH-isolate
Unknown1	1399448037.7 ± 126786681.4	823494814.8 ± 149404994.7
Isoleucine	633666993.8 ± 53579002.6	673010943.2 ± 131461395.0
Valine	630127548.5 ± 41290071.8	562282636.9 ± 92686754.0
Unknown2	5082540668.3 ± 534694576.9	16644394120.7 ± 3904814285.2
Methanol	1797597528.7 ± 174717386.3	6279666594.3 ± 3675257024.5
Succinic acid	2300624134.7 ± 270092102.8	709605160.2 ± 168389974.4
Dimethylamine	578812925.3 ± 95739684.2	437779972.9 ± 128947400.8
Carnitine	3084067966.3 ± 159401799.1	3795174972.0 ± 740774130.0
Trimethylamine N-oxide	3974655442.7 ± 328508341.3	3400014121.3 ± 755438838.7
3,7-Dimethyluric acid	2412312002.7 ± 157833650.2	4685419077.7 ± 1109039639.3
Glucose	327064706.4 ± 30691788.7	77466377.2 ± 4389983.5
Uracil	51778944.5 ± 4193156.1	52496355.1 ± 14638440.4
Epicathecin	153016823.6 ± 14387497.7	65588866.3 ± 12704079.3
Xanthurenate	508870611.7 ± 31153431.9	117346646.2 ± 2389983.7
Histidine	228486258.3 ± 18571747.0	80895677.0 ± 14612836.2
Unknown4	130310772.5 ± 3434231.4	161178156.9 ± 28388734.5
Formate	921754854.8 ± 161323106.3	1302442605.9 ± 476228669.8

### Different patterns of metabolic profile in CH- and TH-isolate

**Fig 5:**
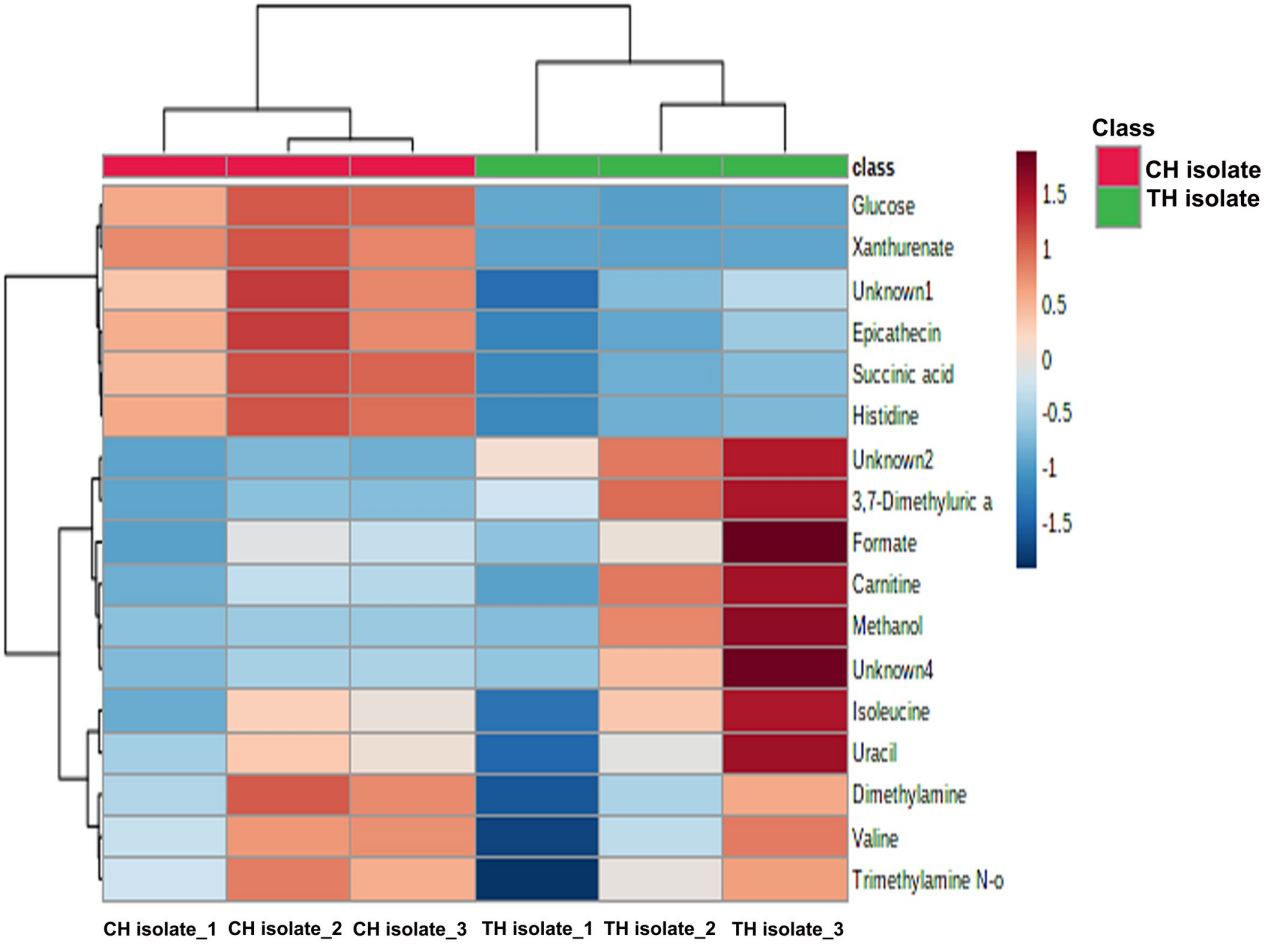
Heatmap of differential metabolites of CH and TH-isolate.

The different metabolites of nine samples could be divided into two main clusters, as illustrated by the hierarchical distance tree, representing the metabolites involved in the phenotype of mushroom types (Fig 5). The result demonstrated that several metabolites are increased in TH-isolate compared with CH-isolate including unknow2, 3,7-dimethyluric acid, formate, carnitine, methanol, and unknown 4. In contrast, some metabolites were decreased in TH-isolate while increased in CH-isolate, such as glucose, xanthurenate, epicatechin, succinic acid, histidine and unknown 1. However, some metabolites, including isoleucine, uracil, dimethylamine, valine, and trimethylamine, were comparably increased in both isolates.

## Discussion

The consumption of functional foods is an increasing trend, especially for natural food sources. Mushrooms are one of them because they are easily prepared, variety-cooked, and lack nutrients [19]. Among mushrooms, bamboo mushrooms are one of the major kinds of edible mushrooms due to their taste, texture, smell, and nutritional ingredients such as fibers, carbohydrates, and proteins [22]. Moreover, it has been used as a medicinal mushroom for a long time in many countries, especially in China, where it is believed to be a therapeutic agent for internal organ recovery [5, 18]. To date, the study of the bamboo mushroom is wider, especially the Chinese strain (*P*. *indusiatus*). Several studies based on the bioactivity of *P*. *indusiatus* extracts, both in vitro and in vivo, have been published during the past ten years. The major constituents are water-soluble polysaccharides, specifically β-d-glucan [4], that affect free radicals *in vitro* and *in vivo* [30–33]. Not only the polysaccharides but the crude extract can be used to mobilize several conditions. Other activities include immunomodulation by inducing macrophage cell proliferation, up-regulating immunostimulatory cytokines [34], and enhancing NK cell-killing activity [35]. Moreover, the crude polysaccharide extract of *P*. *indusiatus* has been shown to exert cytotoxicity to several cancers, such as hepatocellular carcinoma [36], osteosarcoma [37], breast cancer [38], and cholangiocarcinoma [16].

The idea behind this study’s initiative was to investigate the metabolic profile of TH-isolates with different external morphology compared to the well-known species (*P*. *indusiatus*). The short-white skirt bamboo mushrooms (later named TH-isolate) are Thailand’s natural strain that can be found in several areas of the country with a few different characteristics among them [6]. Meanwhile, *P*. *indusiatus* is mostly imported from China. Our expected result from the beginning was that TH-isolate would have the same or similar metabolic profile to *P*. *indusiatus*, which is a well-characterized medicinal mushroom. If it is not much different, the TH strain will be promoted as an economically important plant in the future and will be increasingly farmed in our country. Surprisingly, when the long-white skirt bamboo mushroom (later called CH-isolate) with similar characteristics to *P*. *indusiatus* was taken from the farm and used in our study, the CH-isolate demonstrated distinct species to *P*. *indusiatus* by molecular identification. However, the limitation of this present study was that some information related to the key features, such as spore morphology, odor, and microscopic characteristics, could not be investigated at the time of collection. As we proposed the new species of both isolates, this information will be included in further study to make a definite conclusion.

Even the external morphology of the CH-isolate was similar to *P*. *indusiatus*, but the evolutionary phylogenetic tree suggested that the CH-isolate was closely related to the cluster of *P*. *chiangmaiensis*, *P*. *echinovolvatus*, *P*. *fuscoechinovolvatus*, and *P*. *multicolor* [7] which includes some of Thai isolated species. This result suggested that our CH-isolate will be a different species from *P*. *indusiatus* and will be a novel species. On the other hand, the TH-isolate has a different evolutionary branch that is far away from the clusters of both *P*. *indusiatus* and CH-isolate. It is highly possible that the TH-isolate is the new species even though the ITS-2 score is closely related to *P*. *merulinus*, but the ITS-1 sequence strongly suggested differences. The results from the ITS-1 and ITS-2 regions revealed the same finding that could be used to confirm that the CH and TH-isolates from our study differed from the previously reported species. However, the confirmation of the new species needs to be done in the future because more confirmations are required.

After we found that two isolated bamboo mushrooms have different morphology as well as molecular properties, the nutritional and metabolic profiles of both isolates were investigated. The nutrients of CH-isolate contain higher levels of fat, protein, and ash compared to TH-isolate, whereas carbohydrate content is higher in TH-isolate. However, the overall energetic values of both isolates are almost similar. This result suggested that these mushrooms contain different contents and will be useful for further applications in different ways. The result of the chemical shift, PCA, OPLS-DA, and heatmap demonstrated that most of the metabolites of CH- and TH-isolates were similar but only had some different biochemical constituents. These results confirmed that both isolates could be the source of nutrients, such as essential amino acids (including valine, threonine, histidine, and phenylalanine), non-essential amino acids (such as alanine), and a vast of carbohydrates that would be the food of choice [29, 32]. However, CH-isolate contains a higher level of antioxidants, such as epicatechin, as well as toxic substances, including xanthurenate. On the other hand, TH-isolate contains a higher level of carnitine, which has a biological role in lowering serum lipids [29]. Interestingly, the metabolite named unknown-2 is attractive as it is abundantly produced in only TH-isolate. Unfortunately, this metabolite could not be clarified in this present study. However, the identification of this molecule will be determined later in the further study. Even some metabolites could imply their bioactivities, but the exact bioactivities of the mushroom extracts, as well as bioactive compounds, should be further investigated. However, various factors could affect the variabilities of the NMR study, such as extraction methods, sample stability, interfering compounds in the sample, instrument, data processing and analysis, and biological variability. To minimize the bias and variabilities, we emphasize the importance of rigorous experimental design, standardized protocols, quality control measures, and careful validation of results for addressing the results with reliability and reproducibility of our study [8, 39, 40].

In conclusion, this present study reported the newly isolated bamboo mushrooms from Thailand by morphological and molecular characterizations. The long white skirt (CH-isolate) had an external morphology similar to *P*. *indusiatus* (Chinese bamboo mushroom) but, from our study, had different molecular properties. Meanwhile, the short white skirt (TH-isolate) was totally different and suspected to be a novel species. Moreover, we reported the nutritional and metabolic profiles of these isolated mushrooms, which revealed the differences in their constituents, which could be the source of nutrients but suggested some different bioactivities. Our results could be used for further investigations of beneficial biological and biochemical activities in the future.

## Supporting information

S1 Table The accessions number of bamboo mushroom samples. (DOCX)

S2 Table The raw data of nutritional value (g/100g) of bamboo mushroom samples. (DOCX)
