# Supplementary material for: Morphological characterization, molecular identification, and metabolic profiles of two novel isolated bamboo mushrooms (Phallus sp.) from Thailand
Source: PLoS One. 2024 Oct 24;19(10):e0307157. doi: 10.1371/journal.pone.0307157 (PMC11500925; doi:10.1371/journal.pone.0307157)
Supplement: S1 Table — (DOCX) [file pone.0307157.s001.docx]

**S1 Table. The accessions number of bamboo mushroom samples**

| **Sample ID** | **Accession number** |
| --- | --- |
| CH0306 | LC830479 |
| CH0406 | LC830481 |
| TH0306 | LC830478 |
| TH0406 | LC830480 |
