# Supplementary material for: Morphological characterization, molecular identification, and metabolic profiles of two novel isolated bamboo mushrooms (Phallus sp.) from Thailand
Source: PLoS One. 2024 Oct 24;19(10):e0307157. doi: 10.1371/journal.pone.0307157 (PMC11500925; doi:10.1371/journal.pone.0307157)
Supplement: S2 Table — (DOCX) [file pone.0307157.s002.docx]

**S2 Table. The raw data of nutritional value (g/100g) of bamboo mushroom samples**

| **No.** | **Analytical list** | **Method of Determination** | **Results** | |
| --- | --- | --- | --- | --- |
|  |  |  | **CH-isolate** | **TH-isolate** |
| 1 | Protein (g/100g) | The determination of nitrogen according to kjeldahl in cereals, ASN_3100 by Foss/Kjeltec 8400 | 31.19 | 18.88 |
| 2 | Fiber (g/100g) | AOAC Official Methods of Analysis 21^th^ ed., 2019, method 978.10 by Gerhardt FT12 | 7.60 | 8.85 |
| 3 | Fat. (g/100g) | Extraction of fat in soya beans, ASN_3136 by Foss /2050 | 1.02 | 0.34 |
| 4 | Moisture (g/100g) at 107 ^o^C | Thermogravimetric Analyzer (LECO^®^ TGA-701) | 5.7 | 6.3 |
| 5 | Ash (g/100g) at 600 ^o^C | Thermogravimetric Analyzer (LECO^®^ TGA-701) | 13.1 | 7.2 |
